# Nonoptimal bacteria species induce neutrophil-driven inflammation and barrier disruption in the female genital tract

**DOI:** 10.1016/j.mucimm.2023.04.001

**Published:** 2023-04-29

**Authors:** Marina Costa-Fujishima, Atta Yazdanpanah, Samantha Horne, Alana Lamont, Paul Lopez, Christina Farr Zuend, Kenzie Birse, Morgan Taverner, Riley Greenslade, Max Abou, Laura Noel-Romas, Bernard Abrenica, Oluwaseun Ajibola, Nnamdi Ikeogu, Ruey-Chyi Su, Lyle R. McKinnon, Helen Pymar, Vanessa Poliquin, Alicia R. Berard, Adam D. Burgener, Thomas T. Murooka

**Affiliations:** 1 University of Manitoba, Rady Faculty of Health Sciences, Department of Immunology, Winnipeg, Canada.; 2 Center for Global Health and Diseases, Department of Pathology, Case Western Reserve University, Cleveland, USA.; 3 University of Manitoba, Rady Faculty of Health Sciences, Department of Medical Microbiology and Infectious Diseases, Winnipeg, Canada.; 4 University of Manitoba, Department of Obstetrics, Gynecology, and Reproductive Sciences, Winnipeg, Canada.; 5 National HIV and Retrovirology Labs, JC Wilt Infectious Disease Research Centre, Public Health Agency of Canada, Winnipeg, Canada.; 6 Centre for the AIDS Programme of Research in South Africa (CAPRISA), Durban, South Africa.; 7 Department of Medical Microbiology and Immunology, University of Nairobi, Nairobi, Kenya.; 8 Unit of Infectious Diseases, Department of Medicine Solna, Center for Molecular Medicine, Karolinska Institute, Karolinska University Hospital, Stockholm, Sweden.

## Abstract

Neutrophil recruitment and activation within the female genital tract are often associated with tissue inflammation, loss of vaginal epithelial barrier integrity, and increased risk for sexually transmitted infections, such as HIV-1. However, the direct role of neutrophils on vaginal epithelial barrier function during genital inflammation *in vivo* remains unclear. Using complementary proteome and immunological analyses, we show high neutrophil influx into the lower female genital tract in response to physiological surges in progesterone, stimulating distinct stromal, immunological, and metabolic signaling pathways. However, despite the release of extracellular matrix-modifying proteases and inflammatory mediators, neutrophils contributed little to physiological mucosal remodeling events such as epithelial shedding or re-epithelialization during transition from diestrus to estrus phase. In contrast, the presence of bacterial vaginosis-associated bacteria resulted in a rapid and sustained neutrophil recruitment, resulting in vaginal epithelial barrier leakage and decreased cell-cell junction protein expression *in vivo*. Thus, neutrophils are important mucosal sentinels that rapidly respond to various biological cues within the female genital tract, dictating the magnitude and duration of the ensuing inflammatory response at steady state and during disease processes.

## INTRODUCTION

Inflammation is an important component of protective immunity but must be tightly regulated to limit damage to host tissues. The female genital tract (FGT) has an additional challenge of regulating complex reproductive processes while interfacing with a wide array of external stimuli, including microbes and various physiological and environmental stressors^1^. Incomplete resolution of inflammation, coupled with the inability to restore tissue homeostasis^2^, negatively impacts women’s reproductive health including increased risk of pre-term birth^3^, cervical dysplasia^4^, and sexually transmitted infections (STIs), including HIV-1^5–7^. Unlike other mucosal sites, the structure and function of the FGT are tightly regulated by ovarian sex hormones progesterone (P4) and estradiol (E2) as their levels fluctuate throughout the menstrual cycle. In humans and other mammals, many physiological changes within the FGT can be divided into the E2 dominant follicular phase (proestrus phase in mice), ovulation (estrus), and the P4 dominant luteal phase (metestrus and diestrus phases in mice)^8^. E2 increases during proestrus, reaches a peak during estrus, and gradually wanes if fertilization does not occur, whereas P4 levels peak following ovulation at the diestrus phase in mice^8,9^. These fluctuating sex hormone levels not only influence FGT remodeling but also coordinate unique patterns of epithelial cells, stromal fibroblast, and immune cell function. In the lower FGT, E2 maintains the thickness of the vaginal epithelium and suppresses mucosal immunity to allow fertilization^10,11^ while high levels of P4 initiate an inflammatory response in the vaginal mucosa characterized by leukocyte infiltration that leads to tissue breakdown and bleeding, normally associated with menstruation^12^.

The vaginal microbiota has been implicated in modulating reproductive health, where microbial communities dominated by *Lactobacillus* species are often associated with favorable health outcomes, whereas microbial communities with a higher proportion of facultative and obligate anaerobes and low *Lactobacillus* species are often associated with elevated inflammatory cytokine levels^13,14^ and significantly higher HIV acquisition risk^6^. Strong associations between elevated mucosal inflammatory cytokines with barrier function and immune cell migration have been established in women, of which three are neutrophil-associated proteases (ELANE, MMP9, and lysozyme c)^15–17^, implicating neutrophils as possible drivers of the inflammatory process and vaginal epithelial barrier breakdown in these women. Similarly, changes in the gut microbiota composition can regulate neutrophil function, where signals sensed in local and distal organs modulate their accumulation, survival, and magnitude of the ensuing inflammatory response^18–21^.

Neutrophils rapidly enter tissues after infectious or physical insult, a process that is crucial for the elimination of pathogens and initiating tissue repair^22^. However, sustained and uncontrolled neutrophilic responses can become deleterious to tissues and are often linked to pathology in multiple diseases such as chronic lung disease^23–25^, inflammatory bowel disease (IBD)^26–29^, and more recently, severe SARS-CoV-2 infection^30,31^. However, the interplay between FGT neutrophils, vaginal mucosal barrier integrity, and the microbiome *in vivo* has not been systemically explored.

In this study, we employed complementary immunological and proteomic approaches to assign a mechanistic function to FGT neutrophils at steady state and during bacterial vaginosis (BV)-associated genital inflammation *in vivo*. Progesterone-driven recruitment of neutrophils into the cervicovaginal tissue was transient and contributed minimally to genital epithelial barrier remodeling despite upregulation of biological pathways associated with innate immunity and inflammatory processes. In contrast, intravaginal challenge with BV-associated bacteria species resulted in a rapid and sustained neutrophil recruitment that directly modulated epithelial layer shedding, cell-cell junction disruption, and loss of barrier integrity *in vivo*. Upregulation of biological pathways associated with neutrophil migration and epithelial barrier disruption was similarly observed in cervicovaginal secretions from non-*Lactobacillus* dominant women. Thus, our study provides a mechanistic link between neutrophil activation and vaginal barrier integrity *in vivo* and argues that neutrophils are key mucosal sentinels that respond to both physiological and microbial cues that modulate the FGT immune landscape during health and disease.

## RESULTS

### Biological signatures associated with epithelial layer disruption, innate immune activation, and wound healing are upregulated in mice during the diestrus phase of the estrous cycle

**Fig. 1:**
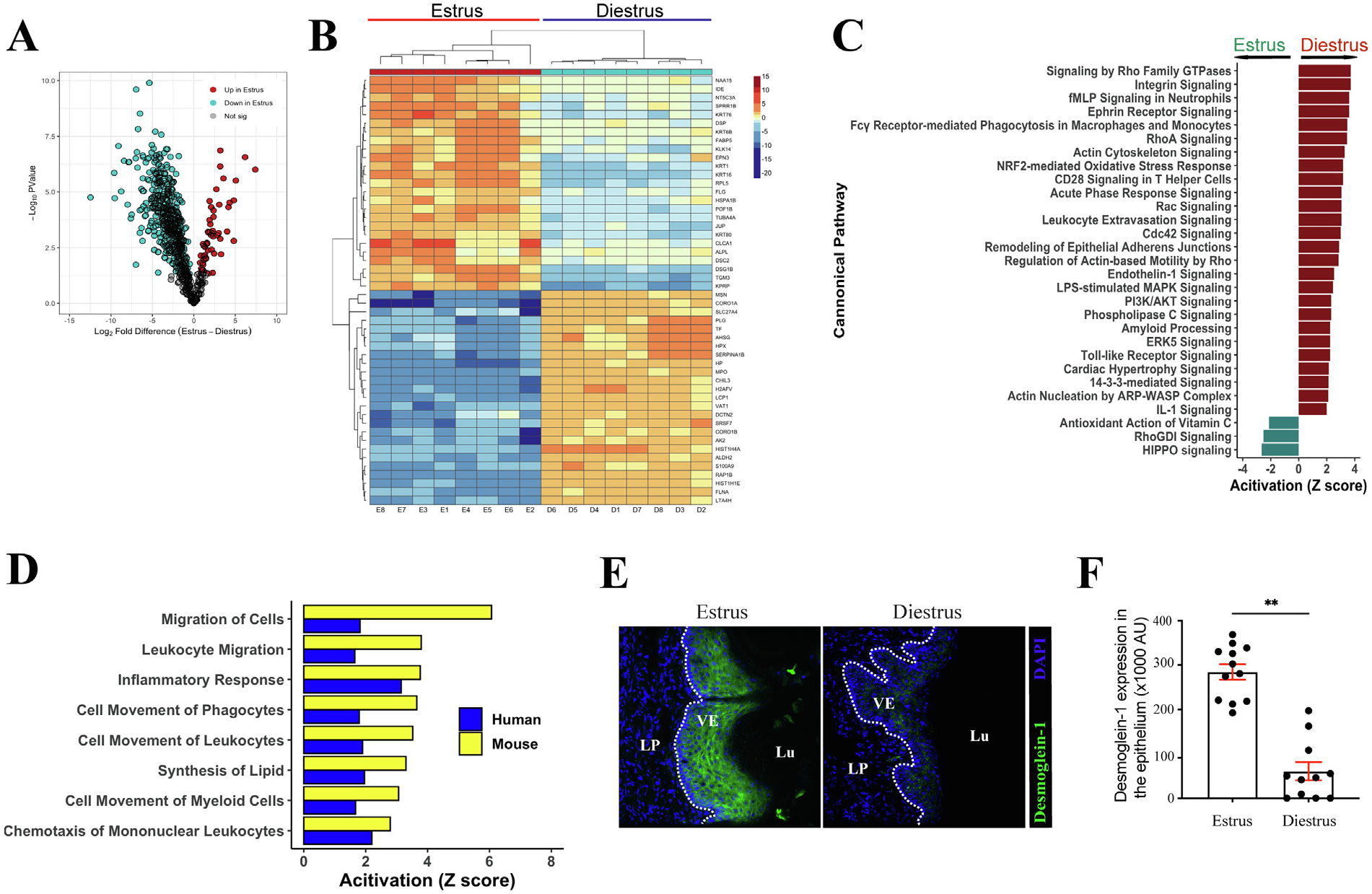
Cervicovaginal lavage protein signatures associated with the estrus and diestrus phases in mice. (A) volcano plot of all proteins identified comparing the estrus-phase protein abundance (positive values) to those measured during the diestrus phase (negative values) *n* = 8 total mice per group; (B) hierarchical clustering of differentially abundant proteins. Proteins that are overabundant are represented in red and those that are underabundant are represented in blue. Estrus phase samples are represented by a red bar at the top of the heat map and diestrus phase samples are represented by a blue bar; (C) canonical pathways significantly enriched during the diestrus and estrus phase of the estrous cycle by IPA analysis. Pathways included have an activation score > |2| and *p* < 0.05; (D) comparative biofunctional associations were determined using Ingenuity Pathway analysis (*p* < 0.007, Right-tailed Fisher’s Exact Test) for both human (Birse et al^32^) and mouse (this paper) with activation z-scores > |2| in mouse dataset and z > |1.5| in human dataset; (E) representative immunofluorescence staining of vaginal tissue from estrus or diestrus mice for DSG1a expression (two independent experiments, *n* = 2 total mice per group shown). DSG1a (green); DAPI (blue). Dotted lines denote the vaginal basal epithelial layer; (F) quantification of DSG1a expression intensity in the VE using ImageJ. Mean ± standard error of mean is shown. **p < 0.01. Each data point represents quantitation from a single image, *n* = 2 total mice per group. A.U = arbitrary units; DSG1a = desmoglein-1; VE = vaginal epithelium; Lu = vaginal lumen; LP = lamina propria.

We first collected cervicovaginal lavage (CVL) from estrus and diestrus mice for mass spectrometry analysis to assess proteome changes between the two phases. We identified a total of 568 unique proteins, of which 50 were found to be differentially abundant between the two phases (≥2-fold change, *p* < 0.05, Mann-Whitney U-test) (Fig. 1A). Some of the overabundant proteins in the estrus phase include desmoglein-1β (DSG1B; 5.1-fold change, *p* = 3.1 × 10^−6^), keratinocyte proline-rich protein (KPRP; 7.4-fold change, *p* = 9.8 × 10^−7^) and junction plakoglobin (JUP; 3.2-fold change, *p* = 1.4 × 10^−7^), whereas overabundant proteins in the diestrus phase included myeloperoxidase (MPO; 7.8-fold change, *p* = 4.5 × 10^−7^), coronin-1A (Coro1A; 12.5-fold change, *p* = 1.8 × 10^−5^), plasminogen (PLG; 8.0-fold change, *p* = 3.7 × 10^−5^), and histone H2A (H2A; 8.4-fold change, *p* = 4.0 × 10^−7^) (Fig. 1B, Supplementary Data File 1). Top canonical pathways enriched in the diestrus phase include remodeling of epithelial adherens junctions, leukocyte migration, and neutrophil chemotaxis (Fig. 1C). High expression of the desmosomal protein, desmoglein-1, was confirmed in the vaginal epithelial cells from estrus, but not diestrus mice by immunohistochemistry, validating our proteome analyses (DSG1B) and previous studies^33–35^ (Figs. 1E and 1F). Together, the murine diestrus phase, characterized by epithelial sloughing^36^, is associated with biological signatures involved in tissue remodeling and leukocyte infiltration, in line with our previous observations from human cohorts (Fig. 1D) and rhesus macaques^32,37^. These data support the use of mouse models to mechanistically examine how environmental cues modulate innate immunity within the lower FGT landscape *in vivo*.

### High neutrophil recruitment into the vaginal mucosa during diestrus

**Fig. 2:**
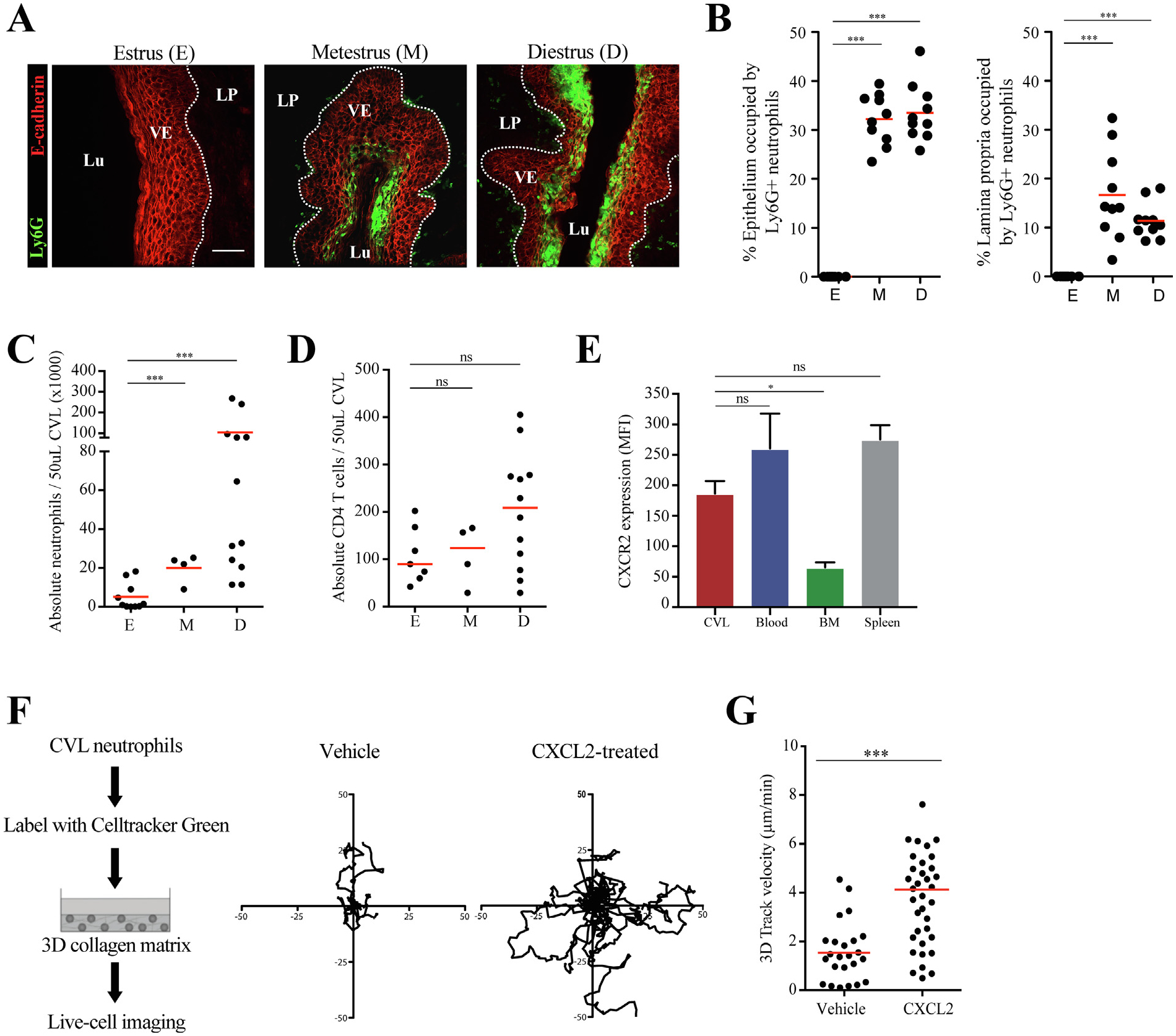
Increased neutrophil influx to the vaginal mucosa during diestrus. (A) representative immunofluorescence staining of vaginal tissues from mice in the estrus, metestrus, or diestrus phase (two independent experiments with *n* = 5 total mice per group). E-cadherin (red); Ly6G+ cells (green). <scale bar = 50 um>; (B) percentage of vaginal epithelium or lamina propria occupied by Ly6G+ neutrophils at different phases of the estrous cycle. Each data point represents quantitation from a single microscopy image, *n* = 5 total mice per group; (C) absolute neutrophil (CD45^+^CD11b^+^Ly6G^hi^); and (D) absolute CD4 T (CD45^+^CD3^+^CD4^+^) cell counts in CVL collected at the indicated estrus phase. Each datapoint represents a mouse, *n* = 4–12 total mice per group; (E) CXCR2 expression (MFI) on neutrophils isolated from CVL, peripheral blood, bone marrow and spleen; (F) CVL neutrophils from diestrus mice were stained with CellTracker green (CMFDA) dye and imbedded into 3D collagen chambers in the presence of 10 ng/ml CXCL2 or vehicle alone (PBS). Cell tracks from a common point of origin are shown; (G) 3D track velocity of individual neutrophils in the presence or absence of CXCL2. Data pooled from two independent experiments, *n* = 2 total mice per group shown. Each datapoint represents a single cell track analysis from 3–5 total movies. All analysis in this figure were performed using unpaired two-tailed t test (Mann-Whitney U-test). *p < 0.05, ***p < 0.001. Red bar indicates mean values. CD = clusters of differentiation; CVL = cervicovaginal lavage; E = estrus; M = metestrus; D = diestrus Lu = vaginal lumen; LP = lamina propria; ns = not significant; VE = vaginal epithelium.

Proteome analysis of mouse CVL indicated that the diestrus phase is associated with innate immune activation and an influx of leukocytes, particularly neutrophils. Immunohistochemical staining of the vagina and ectocervix confirmed high neutrophilic influx into the vaginal mucosa in both the metestrus and diestrus phases but not in estrus, consistent with previous studies^11,38–40^. Neutrophil recruitment was substantial during diestrus, where ~35% of the vaginal epithelium (E-cadherin^+^) was occupied by recruited Ly6G^+^ neutrophils (Figs. 2A and 2B). Remarkably, large numbers of neutrophils were found in clusters along the outer layers of the vaginal epithelium, indicating possible swarming behaviors^41^ as they transit through and exit into the vaginal luminal space. Indeed, large numbers of neutrophils were observed in the CVL during the metestrus and diestrus phases, whereas clusters of differentiation (CD4) T cell numbers were not altered significantly (Figs. 2C and 2D). The chemokine receptor CXCR2 has been shown to direct neutrophil migration into the vaginal epithelium via local expression of ligands CXCL1/2 by epithelial cells during progesterone-dominant diestrus^11,38,42^. Accordingly, vaginal neutrophils expressed CXCR2 at comparable levels to those in peripheral blood and spleen, and higher than those of immature, bone marrow neutrophils (Fig. 2E). To further evaluate CXCR2 functionality, CVL neutrophils were collected, stained with Celltracker green (CMFDA) and embedded into 3D collagen chambers in the presence or absence of CXCL2 (Fig. 2F). Live-cell imaging studies showed that CXCL2 increased neutrophil migration speed and displacement compared to vehicle controls, confirming that CVL neutrophils are viable and actively respond to CXCL2 *ex vivo* (Figs. 2F and 2G).

### Neutrophil recruitment imposes alteration in biological signatures within the lower FGT

**Fig. 3:**
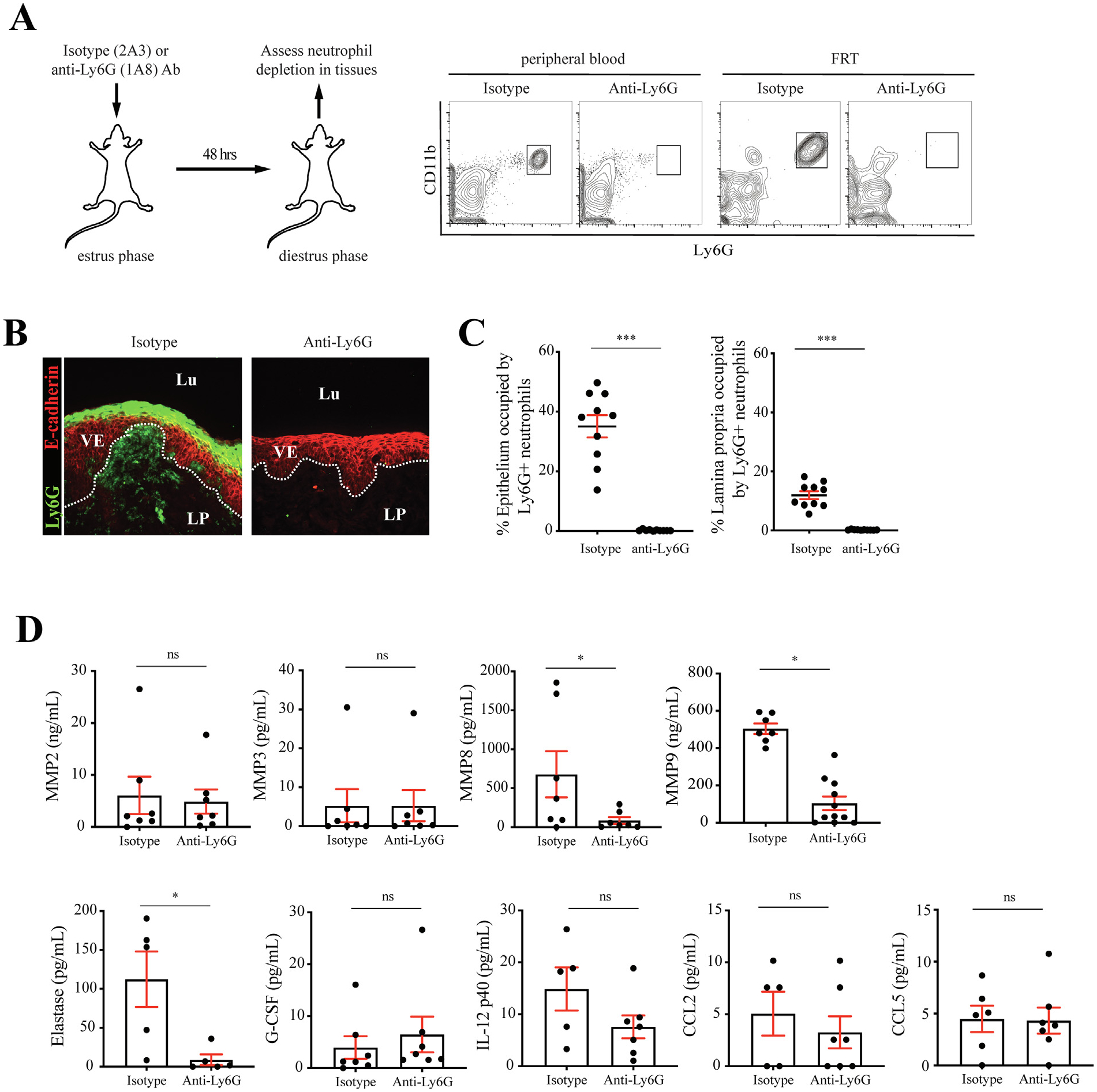
Neutrophil-derived protease expression in CVL at steady-state. (A) experimental schematic for the *in vivo* depletion of neutrophils. Mice were injected with either isotype or anti-Ly6G (clone 1A8) antibody, and neutrophil counts were enumerated in blood (left) and FGT (right) after 48 hours (using clone RB6–8C5); (B) representative immunofluorescence of vaginal tissues stained for E-cadherin (red) and Ly6G^+^ cells (clone: RB6–8C5); (C) percentage of vaginal epithelium of lamina propria occupied by Ly6G^+^ cells after antibody injection. Each data point represents quantitation from a single microscopy image, *n* = 2 total mice per group; (D) cytokine measurements in the CVL of mice after either isotype or anti-Ly6G antibody injection. All analysis in this figure were performed by unpaired two-tailed t test (Mann-Whitney U-test). *p < 0.05; ***p < 0.001. Red bar indicates mean values ± standard error of mean. Each data point represents mean values (performed in triplicates) from a single mouse, *n* = 6–10 mice per group. CD = clusters of differentiation; CVL = cervicovaginal lavage; Lu = vaginal lumen, LP = lamina propria; ns = not significant; VE = vaginal epithelium.

Unlike lymphocytes that use amoeboid migration to squeeze through the extracellular matrix (ECM) in tissues, a key feature of neutrophil tissue migration is the release of ECM-degrading enzymes such as matrix metalloproteinases (MMPs) and serine proteases that disrupt cell-cell junctions during migration^43,44^. We postulated that the large numbers of recruited neutrophils would impose substantial changes to the cervicovaginal mucosal landscape at steady state. We detected elevated expression of MMP2, MMP3, MMP8, and neutrophil elastase (NE) in the CVL of metestrus and diestrus mice (Supplementary Fig. 1). Similarly, the levels of interleukin (IL)-12p40 and CCL5 were higher during metestrus and diestrus, while G-CSF and CCL2 expression levels remained unchanged. To establish the direct contribution of mucosal neutrophils to the secretion of these molecules, mice were injected with either isotype or anti-Ly6G monoclonal antibody (1A8) (Fig. 3A), resulting in neutrophil depletion from peripheral blood and vaginal mucosa after 48 hours that lasted for up to 6 days (Figs. 3B and 3C). We confirmed that MMP8, MMP9, and NE expression in CVL are derived predominantly from mucosal neutrophils (Fig. 3D), possibly contributing to the disassembly of intercellular junctions and the degradation of the extracellular matrix. Global changes in neutrophil-depleted mouse CVL proteome identified a total of 78 differentially abundant proteins compared to non-depleted controls (≥2-fold change, *p* < 0.05, Student’s t test). Database for Annotation, Visualization, and Integrated Discovery (DAVID) analyses identified the expected downregulation of innate immune response pathways (*p* = 0.0056), immune response regulating signaling pathways (*p* = 0.0028), and immune activation pathways (*p* = 6.5 × 10^−4^), whereas biological functions associated with cellular macromolecular catabolism (*p* = 6.08 × 10^−8^), desmosome organization, (*p* = 2.8 × 10^−4^), and morphogenesis of a polarized epithelium (*p* = 2.2 × 10^−5^) were upregulated in mice lacking neutrophils (Supplementary Fig. 2, Supplementary Data File 2). These studies suggest that massive neutrophil influx during diestrus may impose substantial structural changes within the FGT mucosal environment as they enter and exit the vaginal mucosa.

### Neutrophils contribute minimally to vaginal epithelium turnover at steady state

**Fig. 4:**
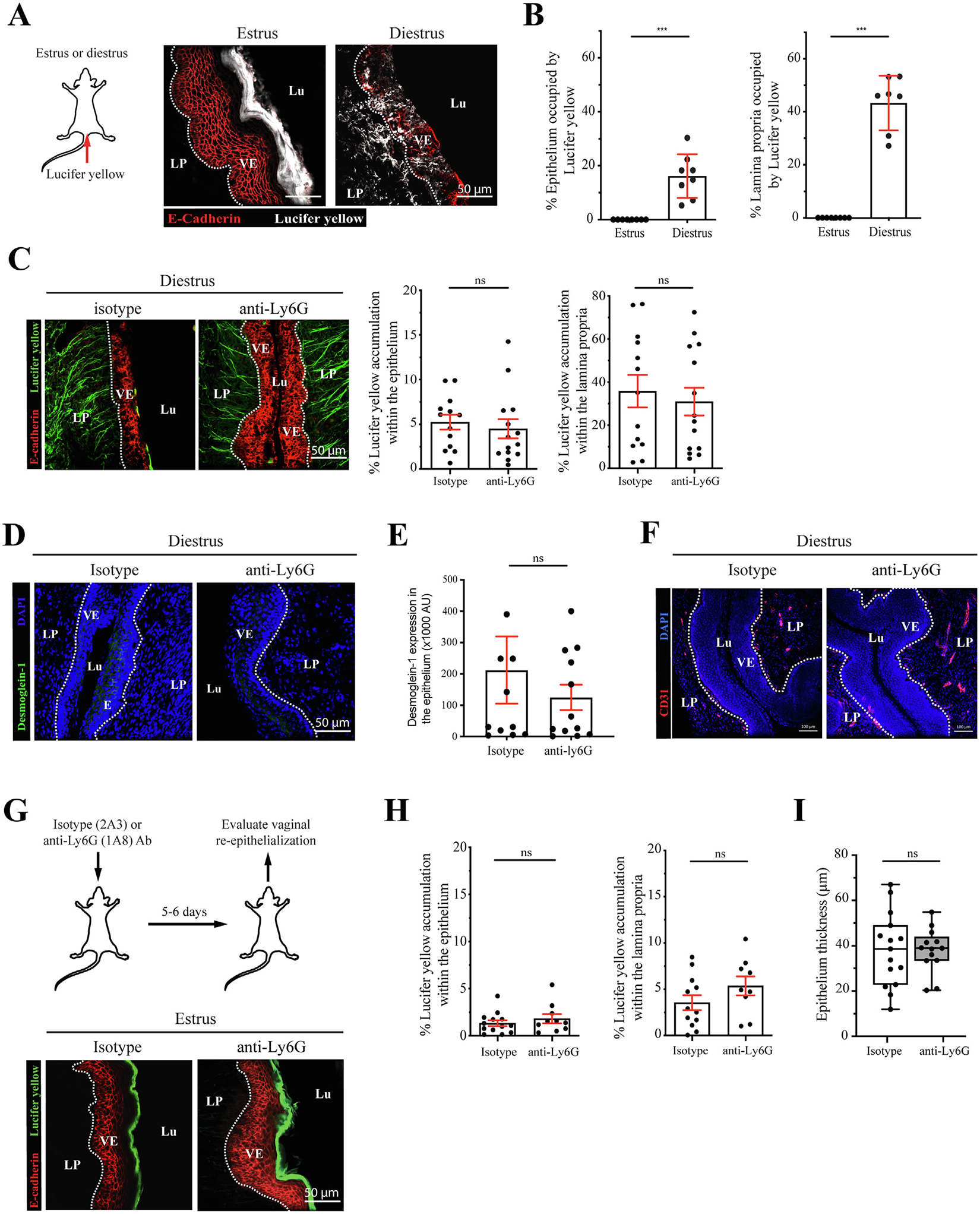
Minimal contribution by recruited neutrophils on vaginal epithelial barrier turnover at steady-state. (A) experimental design. Female Balb/c mice in estrus or diestrus were intravaginally inoculated with LY for 45 minutes before tissue analysis by immunohistochemistry. Right panel: Representative images of LY penetration into the vaginal mucosa in estrus or diestrus mice are shown; (B) percentages of lamina propria or vaginal epithelium occupied by LY are shown. LY dye (white), E-cadherin (red). <scale bar = 50 μm>. Each data point represents quantitation from a single microscopy image, *n* = 2 total mice per group; (C) LY penetration into the vaginal mucosa after the indicated antibody injection (2 days). Each data point represents quantitation from a single microscopy image, *n* = 2 total mice per group. The percentage of lamina propria or vaginal epithelium occupied by LY for each condition is shown. Mean ± standard error of mean. <scale bar = 50 μm>; (D) desmoglein-1 expression within the vaginal epithelium in mice injected with either isotype or anti-Ly6G antibody. Dotted white line indicate the basal layer of the epithelium. <scale bar = 50 μm>; (E) quantification of DSG1a expression intensity in the vaginal epithelium using ImageJ. Each data point represents quantitation from a single microscopy image, *n* = 2 total mice per group; (F) staining for CD31 after antibody injection in diestrus mice; (G) schematic representation of female Balb/c mice injected with either anti-Ly6G or isotype antibody and assessed for vaginal re-epithelization after 5 days. The degree of LY penetration into the vaginal mucosa in estrus mice is shown; (H) percentage of lamina propria or vaginal epithelium occupied by LY. Each data point represents quantitation from a single microscopy image, *n* = 2 total mice per group; (I) vaginal epithelial thickness. All analysis for this figure were performed by unpaired two-tailed t test (Mann-Whitney U-test). ***p < 0.001. Red bar indicates mean values ± standard error of mean. AU = arbitrary units; CD = clusters of differentiation; CVL = cervicovaginal lavage; Lu = vaginal lumen; LP = lamina propria; LY = lucifer yellow; ns = not significant; VE = vaginal epithelium.

The transition from estrus to diestrus is accompanied by the shedding of the outer layers of the squamous vaginal epithelial layer in mice. We measured the integrity of the vaginal epithelium during this transition by intravaginally administrating lucifer yellow (LY), a small molecular weight fluorescent dye (0.46kDa), for 45 minutes under isoflurane anesthesia prior to immunohistochemical analysis (Figs. 4A and 4B). As expected, the thinning of the vaginal epithelial lining during diestrus resulted in a high dye influx into the lamina propria (~40% of the lamina propria was LY^+^), in contrast to the robust protection observed in estrus. We next examined whether neutrophils were directly contributing to epithelial barrier shedding by performing *in vivo* depletion and LY accumulation studies, as described above. Interestingly, the absence of neutrophils did not have a measurable impact on dye permeability across the vaginal epithelium at diestrus (Fig. 4C). Although previous studies have reported a direct role of neutrophils in tissue remodeling in the upper FGT^45^, no differences in desmoglein-1 expression (Figs. 4D and 4E) or neo-vascularization after neutrophil depletion was noted in the vagina or ectocervix (Fig. 4F). We also observed no measurable defects in FGT re-epithelialization, where neutrophil-depleted animals were followed for one complete estrous cycle prior to LY challenge and epithelium thickness measurements (Figs. 4G–I). These studies indicate that while neutrophil influx and the release of ECM-modifying enzymes are distinct features of the cervicovaginal tissue at diestrus, they did not participate in epithelial layer shedding or re-epithelialization at steady state in the lower FGT.

### FGT neutrophils drive epithelial barrier dysfunction during BV-associated microbial challenge

**Fig. 5:**
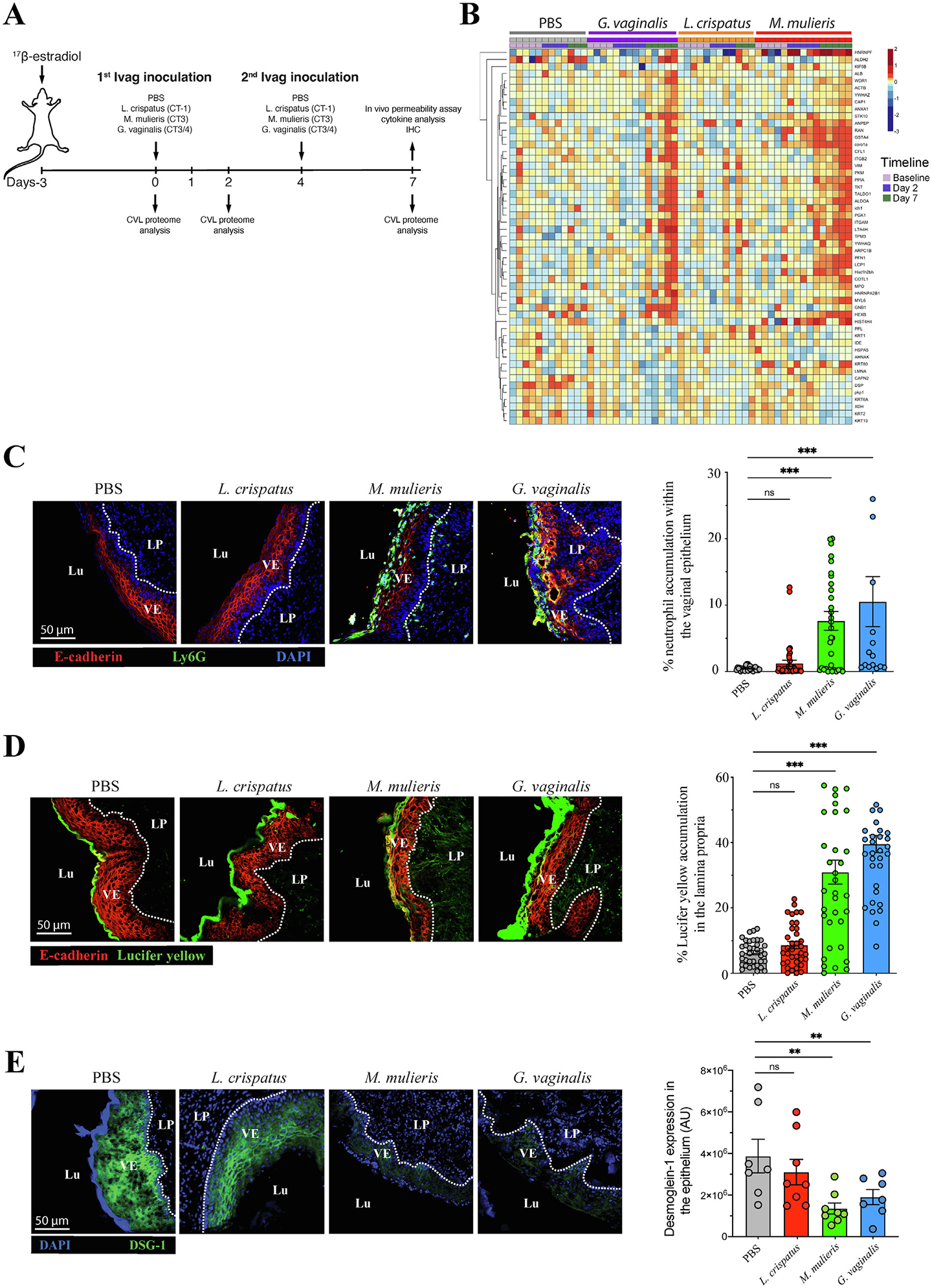
Intravaginal challenge with bacterial vaginosis-associated bacteria results in sustained neutrophil recruitment, inflammation, and vaginal barrier disruption. (A) Schematic representation of intravaginal bacterial challenge studies in Balb/c mice. CVL and tissues were collected throughout the study for analysis.;(B) hierarchical clustering of differentially abundant proteins identified in CVL of mice at baseline and at day 2 and 7 after intravaginal bacterial challenge. Proteins that are overabundant are represented in red and those that are underabundant are represented in blue; (C) representative images of the vaginal tissue at day 7 post-challenge to visualize infiltrating neutrophils (two independent experiments, *n* = 3 total mice per group shown). Ly6G^+^ (green); E-cadherin (red); DAPI (blue); Right panel: Percentage of lamina propria or vaginal epithelium occupied by Ly6G^+^ cells. Each data point represents quantitation from a single microscopy image, n = 3 total mice per group; ***p < 0.001. Red bar indicates mean values ± SEM. <scale bar = 50 um>; (D) representative images of the vaginal tissues after bacterial challenge. Lucifer yellow (green); E-cadherin (red). Right panel: Percentage of lamina propria occupied by lucifer yellow. Each data point represents quantitation from a single microscopy image, data pooled from two independent experiments, *n* = 3 total mice per group shown. ***p < 0.001. Red bar indicates mean values ± SEM; E, DSG-1 expression at day 7 post-bacterial challenge. Dotted line depicts the basal layer of the vaginal epithelium. Quantification of DSG-1 expression intensity in the vaginal epithelium using ImageJ is shown. Each data point represents quantitation from a single microscopy image, *n* = 3 total mice per group. Mean ± SEM is shown. AU = arbitrary units; CD = clusters of differentiation; CVL = cervicovaginal lavage; DSG-1 = desmoglein-1; FC = fold change; Lu = vaginal lumen; LP = lamina propria; SEM = standard error of mean; VE = vaginal epithelium.

**Fig. 6:**
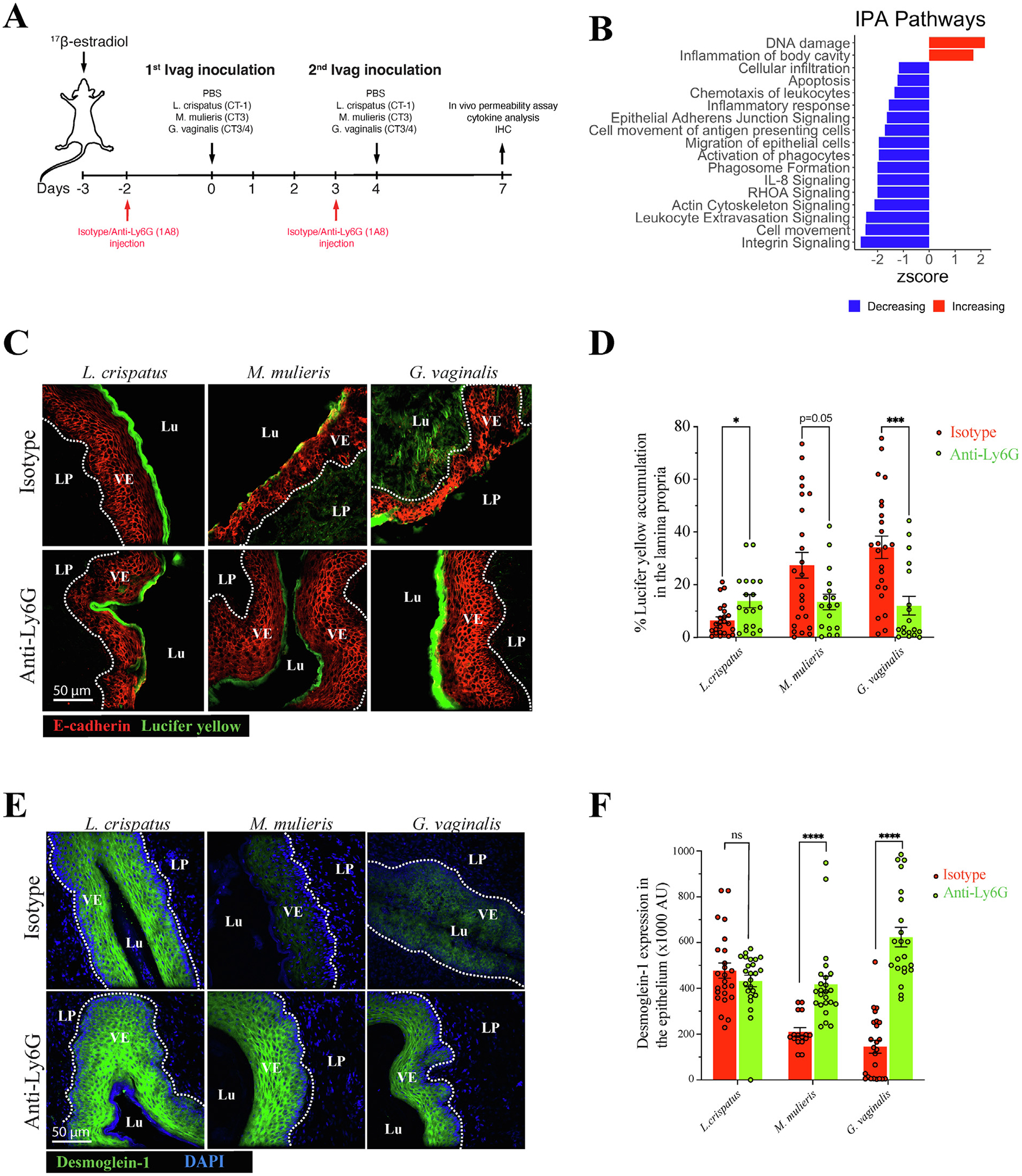
Recruited neutrophils directly disrupt the vaginal epithelium in the presence of BV-associated bacteria *in vivo*. (A) Schematic representation of intravaginal bacterial challenge studies *in vivo*; (B) canonical pathways significantly enriched in CVL after intravaginal challenge with BV-associated bacteria in the absence of neutrophils. Pathways included have an activation score > |2| and *p* < 0.05; (C) representative images of vaginal tissue after the indicated bacterial challenge in the presence or absence of neutrophils (two independent experiments, *n* = 3 total mice per group shown). Lucifer yellow dye (green); E-cadherin (red). <scale bar = 50 um>; (D) percent lucifer yellow penetration into the lamina propria. Each data point represents quantitation from a single microscopy image, *n* = 3 total mice per group. *p < 0.05, ***p < 0.001; (E) representative image of the vaginal mucosal sections after staining for DSG-1 at day 7 post-bacterial challenge (two independent experiments, n = 3 total mice per group shown). Dotted line depicts the basal layer of the vaginal epithelium; (F) quantification of DSG-1 expression intensity in the vaginal epithelium using ImageJ is shown. Each data point represents quantitation from a single microscopy image, *n* = 3 total mice per group. Mean ± standard error of mean is shown. AU = arbitrary units; BV = bacterial vaginosis; CD = clusters of differentiation; CVL = cervicovaginal lavage; DSG-1 = desmoglein-1.

While neutrophils did not have a measurable impact on epithelial barrier turnover at steady state, we next asked whether neutrophil recruitment and function were altered in the presence of BV-associated bacteria. To test this, estrogenized mice were intravaginally inoculated atraumatically with either phosphate-buffered saline (PBS), *Lactobacillus crispatus*, *Mobiluncus mulieris*, or *Gardnerella vaginalis*, as outlined in Fig. 5A. *L. crispatus* dominance of the vaginal flora is strongly associated with positive health outcomes, and their vaginal inoculation has been reported to improve endometrial injury in mice^46^ or *G.vaginalis* induced epithelial cell exfoliation^47^, whereas *M. mulieris* and *G. vaginalis* are associated with bacterial vaginosis (BV)^13,48^, the latter causing clinical BV-like symptoms in mice^49,50^. Synchronizing mice in the estrus phase removed the confounding effects of sex hormones on neutrophil recruitment and other cellular functions in the FGT^14^, and normalized barrier integrity and thickness across all conditions. 16S ribosomal RNA (rRNA) sequencing of CVL samples collected at various times post-challenge confirmed the presence of inoculated bacteria (Supplementary Fig. 3A), and mass spectrometry analysis at day 7 post-challenge indicated significant upregulation of proteins involved in neutrophil activation and migration (Coro1a, MPO, ITGM-2) in the presence of *M. mulieris* or *G. vaginalis*, but not PBS or *Lactobacillus crispatus*, consistent with biological pathways upregulated in CVL of women with elevated inflammatory cytokine expression^15–17,51^ (Fig. 5B, Supplementary Fig. 3B, and Supplementary Data File 3). Neutrophil recruitment into the vaginal epithelium and lamina propria was confirmed by immunohistochemistry after *M. mulieris* and *G. vaginalis* challenge as early as day 2 (Supplementary Fig. 3C) and continued to accumulate at day 7 post-challenge, but not observed in PBS and *L. crispatus* challenged mice (Fig. 5C). We detected higher levels of neutrophil-derived ECM-modulating proteases after challenge with *M. mulieris* and *G. vaginalis*, although differential expression levels were observed between the two groups (Supplementary Fig. 3E). We next explored the *in vivo* consequences of different vaginal microbial challenges and neutrophil recruitment on epithelial barrier integrity, using LY as readout. Strikingly, substantial leakage of the dye into the lamina propria was observed in the vaginal mucosa of mice challenged with *M. mulieris* and *G. vaginalis* compared to control mice (Fig. 5D). We also observed strong downregulation of DSG-1 expression within the vaginal epithelium of these mice (Fig. 5E). Significant thinning of the vaginal epithelium was also evident, suggesting an increase in epithelial cell exfoliation, a phenotype clinically used to diagnose women with BV^52,53^ (Supplementary Fig. 3D). Notably, increased barrier breakdown was also observed in mice challenged with *M. mulieris* and *G. vaginalis* synchronized to diestrus compared to control mice, but here *L.crispatus* also induced similar barrier loss (data not shown). Finally, we examined the direct role of neutrophils on epithelial barrier loss in the presence of BV-associated bacterial species by depleting circulating neutrophils *in vivo*, 48 hours prior to bacterial challenge (Fig. 6A). Ingenuity Pathway Analysis (IPA) analysis of CVL collected at day 7 after bacterial challenge in the context of neutrophil depletion revealed a reduction in epithelial adherens junction signaling, migration of epithelial cells, and integrin signaling (Fig. 6B). Interestingly, we observed a near complete restoration of epithelial barrier function in *G. vaginalis* and *M. mulieris* (p = 0.05) challenged mice when neutrophils were absent (Figs. 6B and 6C). Similarly, DSG-1 expression was completely restored upon neutrophil depletion, indicating direct modulation of vaginal epithelial barrier integrity by these cells in the presence of BV-associated bacteria. Collectively, these data demonstrate that rapid and sustained neutrophilic responses to inflammatory bacterial challenge drive substantial vaginal barrier disruption in a manner that is distinct from their physiological role at steady state.

### Epithelial dysfunction and neutrophil activation are associated with a non-*Lactobacillus* dominant (nLD) microbiome in women

**Fig. 7:**
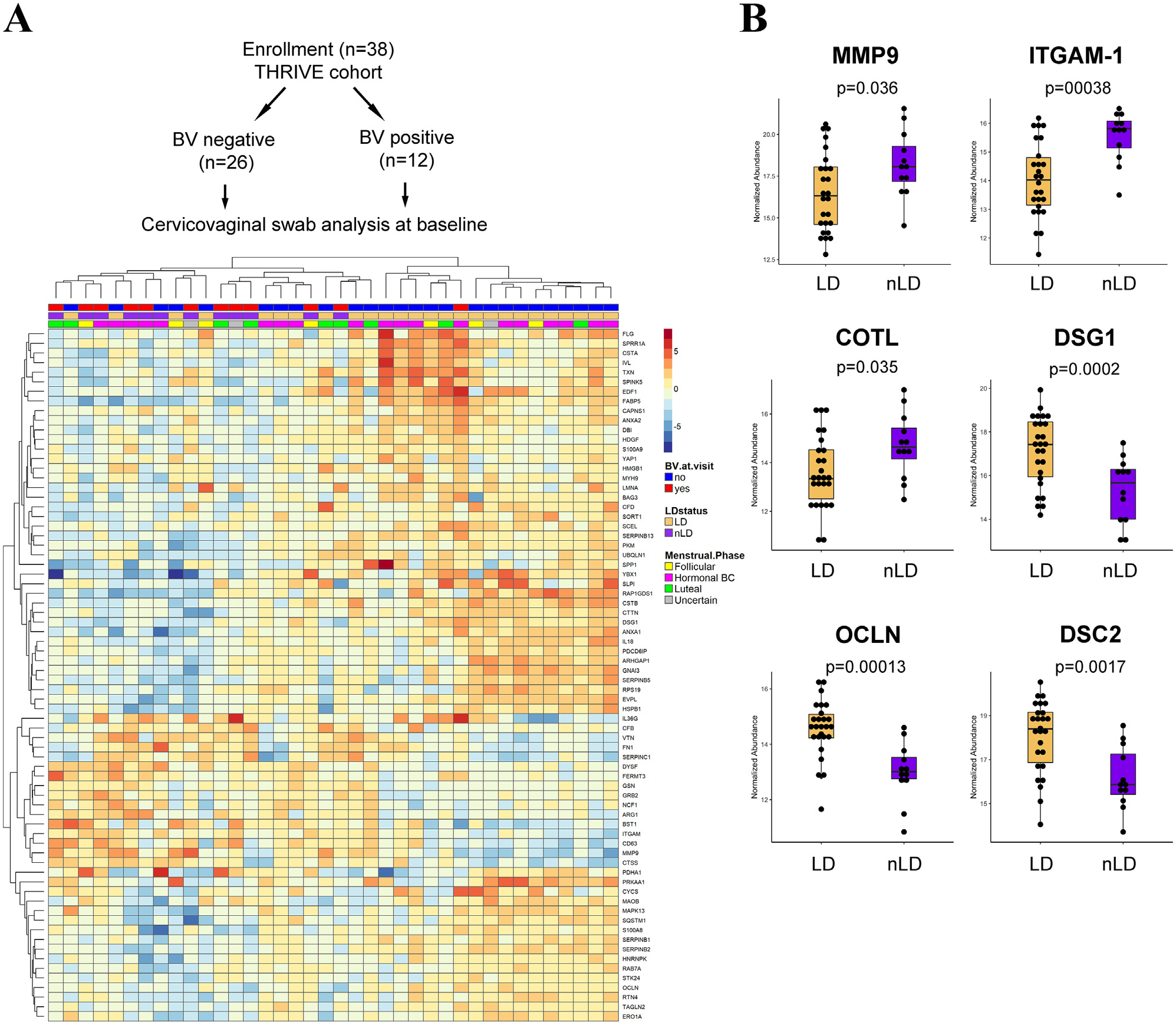
Neutrophil migration and epithelial barrier disruption pathways cluster in women with non-LD vaginal microbiome. (A) Heat map of differentially abundant proteins (*p* < 0.05) at baseline between LD and non-LD women from the THRIVE cohort (n = 38) that are related to neutrophil migration/function and epithelial barrier maintenance pathways. Proteins that are overabundant are represented in red and those that are underabundant are represented in blue; (B) differential protein abundance between LD and non-LD cervicovaginal lavage and their associated *p* values are shown. Mean ± standard error of mean is shown. Unpaired two-tailed t test (Mann-Whitney U-test). BV = bacterial vaginosis; DSG-1 = desmoglein-1; LD = *Lactobacillus dominant*; nLD = non-*Lactobacillus dominant*.

We conducted proteomic and 16S rRNA sequencing analysis of baseline cervicovaginal swab samples collected from 38 participants, of which 12 were diagnosed with BV and 25 without BV at enrollment as part of the The study of Host-bacterial Relationships and Immune function in different Vaginal Environments (THRIVE) cohort. Women in this analysis had an average age of 31.7 (range 20–49), and self-reported as Caucasian (76%, *n* = 28), First Nations (13%, *n* = 5), and other ethnicities (11%, *n* = 4). There were no clinical or demographic differences between the groups, including age, birth control use, antibiotic use, and STI history (Supplementary Table 1). All women were evaluated for STIs at clinical visits and were negative for chlamydia, gonorrhea, and HIV at the time of sampling. For greater statistical power, women were characterized into two major microbiome groups: *Lactobacillus dominant* (LD) and non-*Lactobacillus dominant* groups (nLD). Of the unique proteins differentially expressed between the two groups, we compared differences in neutrophil and epithelial functional pathways at the proteome level using IPA (Fig. 7A). Significant upregulation of neutrophil-associated proteins MMP-9 (*p* = 0.036), ITGAM-1 (*p* = 3.8 × 10^−4^) and COTL (*p* = 0.035), and downregulation of epithelial cell-cell junction proteins desmoglein-1 (DSG-1; *p* = 2.0 × 10^−3^), desmocollin-2 (DSC-2; *p* = 1.7 × 10^−3^), and occludin (OCLN; *p* = 1.3 × 10^−4^) were detected in cervicovaginal secretions of nLD women (Fig. 7B). Subgrouping LD women as either *L. iners* dominant (*n* = 12) and *Lactobacillus* (non-*iners*) species dominant (*n* = 14) prior to comparison against the nLD group resulted in similar observations to that of LD/nLD comparisons (data not shown). We also followed five participants that shifted from an nLD to LD vaginal microbiome 1 month after treatment, which was accompanied by statistically significant reductions in MMP-9 and COTL1 expression and a downward trend (*p* = 0.064) in ITGAM-1 expression in CVL (Supplementary Fig. 4). We observed an upward trend for both DSG1 and OCLN expression upon treatment, although these observations did not reach statistical significance in this small subset of participants. These studies indicate an inverse relationship between neutrophil influx and epithelial barrier function in nLD women and are in line with murine studies that directly link BV-associated anaerobes with genital barrier dysfunction and neutrophil activation *in vivo*.

## DISCUSSION

The mucosal lining of the FGT provides a robust physical barrier that protects the host from the external environment. When the integrity of this epithelial barrier is compromised, women are more susceptible to vaginal infection^51,54^, underscoring the notion that an intact FGT barrier is a crucial component of vaginal mucosal health. Here, we performed complementary proteomic and immunological analyses to assign a mechanistic role to neutrophils in the lower FGT at both steady state and during BV-associated genital inflammation *in vivo*. High progesterone levels led to rapid but transient neutrophil recruitment into the cervicovaginal tissues that were accompanied by cell-cell junction disruption, activation of innate immunity, and elevated ECM-modifying protease expression in vaginal secretions. However, neutrophils at this stage did not impose measurable changes to vaginal epithelial integrity, shedding, or re-epithelialization, suggesting that neutrophil responses *per se* do not contribute to vaginal epithelial remodeling in the lower FGT during the transition to diestrus. In contrast, a vaginal challenge with BV-associated bacteria species led to rapid and sustained neutrophil recruitment that directly modulated epithelial layer shedding, cell-cell junction protein expression, and barrier integrity *in vivo*. Similar upregulation of neutrophil migration and epithelial barrier disruption-related pathways were observed in cervicovaginal secretions from nLD women in the THRIVE cohort, underscoring the role of neutrophils as mucosal sentinels that respond to both physiological and microbial cues during health and disease within the lower FGT.

During the menstrual cycle, the vaginal epithelium undergoes substantial remodeling in response to fluctuating levels of the ovarian sex hormones estradiol and progesterone^32,38^. Previous studies showed that neutrophil influx to the vaginal mucosa in both humans and mice is contingent on the ovarian cycle and occurs when progesterone levels are high^11,38,55^. In humans, neutrophil influx into the uterus is observed before the onset of menses following an increase in IL-8 secretion by the uterine endometrium^56^. Similarly, neutrophils migrate into murine uterine and vaginal tissues post-ovulation in response to surges in CXCL1 (the mouse homolog of human IL-8) by vaginal epithelial cells responding to progesterone^11,38,45,57^ and it is possible that epithelial sloughing reinforces their recruitment^58^. Upon exiting the microcirculation, neutrophils secrete ECM-modifying enzymes, such as serine proteases and MMPs, to facilitate their polarization and migration within interstitial tissue^59^. While many myeloid cells can produce these proteases, we identified MMP8, MMP9, and NE (ELANE) as those that were secreted exclusively by vaginal neutrophils *in vivo*. Following movement through the interstitium, neutrophils reach the subepithelial space and cross the multilayered vaginal epithelium before reaching the stratum corneum and luminal space^60,61^. Neutrophil movement across the epithelium occurs along the basolateral epithelial membrane through the paracellular space, where multiple adhesive interactions between epithelial cells and neutrophils take place, including integrins ITGAM-1 (or CD11b) and ITGB-2 (or CD18)^60^. Studies showed that these interactions, along with the release of matrix-degrading proteases, can influence epithelial barrier integrity^62^. We have previously shown that the progesterone-dominant luteal phase is associated with biological signatures associated with immune cell migration/adhesion, inflammation, and epithelial barrier remodeling^32^, which are in line with findings in mice reported here. However, we did not observe any measurable contribution by neutrophils on epithelial barrier remodeling during diestrus, nor did they contribute to re-epithelialization of the vaginal barrier. These studies indicate that the structural integrity and remodeling of the vaginal epithelium is regulated primarily by sex hormone-derived signaling in epithelial cells, where conditional ablation of estrogen receptor from epithelial cells have been shown to directly deteriorate the vaginal epithelium^10,63^. Rather, neutrophilic influx into the vaginal mucosa during diestrus seems to play an important role in innate protection against incoming sperm and sexually transmitted infections^64–66^. For example, progesterone-driven neutrophilic recruitment helps clear *Candida albicans* infection and reduces inflammation driven by chemokines CXCL1/2^11^. Vaginal neutrophils may also have important homeostatic functions by keeping commensal bacterial communities in check during transitions from estrus to diestrus^67^ or contributing to other wound healing processes^68^. Consistent with this notion, bacterial challenge during diestrus induced an inflammatory process that contributed to loss of barrier integrity. Collectively, our data indicate that despite neutrophils carrying an arsenal of proteases and oxidants that have the capacity to cause considerable tissue damage, their transient influx into the vaginal mucosa contributes little to tissue remodeling during physiological epithelial turnover, challenging the prevailing notion that tissue neutrophils induce collateral tissue damage and inflammation regardless of the activating stimuli.

The role of neutrophils changes drastically during microbial insult, where they orchestrate “swarming” behaviors that result in considerable tissue damage and drive a chronic inflammatory state^69–71^. BV is an inflammatory condition characterized by an outgrowth of nonoptimal anaerobes, including *Gardnerella*, *Mobiluncus, and Prevotella* spp., that are associated with high inflammatory cytokine levels^13,14^ and increased risk of pre-term birth^3^, cervical dysplasia^4^, and STI infection, including HIV-1^5,6^. Strong linkages between elevated mucosal inflammatory cytokines with barrier function and immune cell migration have been established in women, of which three are neutrophil-associated proteases (ELANE, MMP9, and lysozyme c)^15–17^, implicating neutrophils as possible drivers of the inflammatory process. Our proteome analysis of women in the THRIVE cohort also implicated neutrophils as key modulators of BV-associated mucosal inflammation and vaginal epithelial layer remodeling. Upon treatment, women who shifted from an nLD to LD vaginal microbiome displayed reduced proteomic signatures associated with neutrophil migration and an increasing trend in cell-cell junction protein expression. In line with these observations, intravaginal challenge of mice with *M. mulieris* and *G. vaginalis* upregulated biological signatures associated with neutrophil migration (ITGB2, MMP9), leukocyte extravasation and integrin signaling, and large neutrophilic influx was accompanied by significant loss of DSG-1 expression in the vaginal epithelium *in vivo*. Phenotypic changes in vaginal neutrophils were observed in nLD women including delayed apoptosis and disruption of epithelial barrier function in the presence of BV-associated bacteria *in vitro*^72^. The higher presence of neutrophil extracellular traps in vaginal discharge from BV women is also indicative of increased neutrophil influx and activation^73^. Direct binding of neutrophils to desmosomes during transepithelial migration has not been reported, but neutrophil-derived proteases, such as MMP9, can directly cleave DSG-1^74–76^. The cleavage of DSG-1 actively regulates epithelial cellular processes such as apoptosis and differentiation, thus directly influencing epithelial homeostasis and function^77^. A recent study showed that antigen-presenting cells, and not neutrophils, were key modulators of CT3/4-associated inflammatory responses in the lower FGT^14^. Whether differences in neutrophil activation states/subsets^78^ or differences in cohort demographics between the two studies contributed to these conflicting data is unknown and is the subject of our ongoing investigation. Notably, we observed a modest but statistically significant increase in vaginal epithelial permeability in *L. crispatus*-challenged mice in the absence of neutrophils. It is possible that *L. crispatus* has limited capacity to disrupt the vaginal epithelium themselves as they are not a major component of the mouse vaginal micro-biome, which is typically populated by *Staphylococcus*, *Enterococcus*, and to a lesser extent, *Lactobacillus*^79,80^. Alternatively, that neutrophil crosstalk with other immune cells to promote optimal barrier function is required, such as T helper (Th)17 cells^81,82^. We also do not rule out the contribution of anaerobes and their products negatively impacting vaginal epithelial cell function directly^53,83,84^, based on the fact that barrier disruption is still observed in the complete absence of neutrophils in some mice. Finally, while our studies have largely focused on how neutrophils interface with the multilayered squamous epithelium of the vagina and ectocervix, it would be important to determine whether similar responses are observed across the columnar epithelium of the endocervix and endometrium.

It is becoming clear that an inflammatory process should be viewed as a spectrum^85^. Our data demonstrate that while neutrophils are equipped with an array of pro-inflammatory proteases and oxidants, their recruitment and release of tissue-degrading enzymes do not necessarily initiate deleterious inflammatory processes. Physiological neutrophil influx likely contributes to FGT homeostasis by conducting mucosal immunosurveillance at a time when the vaginal epithelium is most vulnerable. Here, any collateral damage is minimized by their rapid exiting of tissues that is tightly controlled by sex hormones. In the presence of BV-associated anaerobes, however, we found sustained neutrophil recruitment and activation that seems to drive a prolonged inflammatory process that leads to substantial epithelial barrier disruption. In this scenario, the release of neutrophil-derived proteases and pro-inflammatory mediators may have a significant negative impact on vaginal barrier integrity *in vivo*. How similar panels of ECM-modifying proteases could have differential effects on the FGT between the two scenarios is unclear and a limitation of this study, but possibilities include modulation of biological activity based on proteolytic cleavage efficiency or the abundance of bodily fluid/tissue inhibitors such as α2-microglobulin and TIMPs, particularly for MMPs^86,87^. Another limitation is that we have not identified which bacteria or host-derived factors/metabolites are responsible for neutrophil recruitment after BV-associated bacterial challenge, and whether blocking these interactions can dampen the ongoing inflammatory process. Our studies argue that distinct physiological and mucosal environmental cues dictate the magnitude and duration of the ensuing neutrophil response within the FGT, some of which are associated with negative health outcomes. The impact of neutrophil-driven loss of vaginal epithelial integrity on HIV-1 transmission, and how this is potentially counterbalanced by their reported ability to eliminate the virus through NETosis formation^88–90^, is the subject of our ongoing research efforts.

## METHODS

### Cervicovaginal lavage collection

CVL were collected from naïve female BALB/c mice (6–8 weeks old; Charles River) and maintained at the Central Animal Care Services, University of Manitoba. Mice were anesthetized with isoflurane and CVL was collected by washing the vaginal tract with 70 ul of sterile PBS. The mouse estrous phase was determined by vaginal cytology^91^. CVLs were centrifuged for 10 minutes at 4°C and the cell-free supernatant was stored at −80°C for further analysis. All animal use protocols were approved by the Animal Care Committee at the University of Manitoba.

### Flow cytometry analysis

Mice FGT were harvested and placed in PBS solution containing 500 ul Collagenase D (Cat# 11088866001, Sigma-Aldrich, USA) and 20 ul of 2M Calcium Chloride. Tissues were minced with scissors and placed in a 37°C water bath for 30 minutes. Tissues were filtered through a 40-um nylon mesh strainer, and single cell suspensions washed by centrifugation and resuspended in PBS at a final concentration of 1 × 10^6^ cells/ml. Cell suspensions from CVL, peripheral blood, spleen, bone marrow, and FGT were pretreated with 5 ul/ml Fc blocker (Cat #101301, Biolegend) for 10 minutes at 4°C and stained with the following surface antibodies: CD3 (clone 17A2, Biolegend), CD45 (clone 30-F11, Biolegend), Ly6G (clone 1A8, Biolegend), CD11b (clone M1/70, biolegend), CD182 (clone SA044G4, Biolegend) for 20 minutes at 4°C and washed once. Flow cytometry data were acquired using the LSR II (BD Biosciences) and analyzed with FlowJo 10.3 (Ashland, OR).

### Bacterial cultures

*Lactobacillus crispatus* type strain DSM 20584 (Leibniz DSMZ) was grown on De Man, Rogosa and Sharpe agar (MRS; National Microbiology Lab media prep). After 48 hours, 1–2 colonies from the plate were suspended in 2 ml NYC III broth (ATCC 1685) and grown at 37 °C for 24 hours under anaerobic conditions, achieved using BD GasPak Anaerobic Sachets (Becton-Dickinson). *Gardnerella vaginalis* type strain ATCC 14018 (ATCC Cedarlane) was grown on tryptic soy agar with 5% sheep’s blood (TSAB; National Microbiology Lab media prep). After 48 hours, a loopful of growth from the plate was suspended in 2 ml NYC III broth (ATCC 1685) and grown at 37 °C for 24 hours under anaerobic conditions, achieved using BD GasPak Anaerobic Sachets (Becton-Dickinson). *Mobiluncus mulieris* type strain DSM 25311 (Leibniz DSMZ) was grown on tryptic soy agar with 5% sheep’s blood (TSAB; National Microbiology Lab media prep) for 96 hours. Plate growth was harvested into 2 ml of NF broth, an in-house mixture of 1:1 NYC III (ATCC 1685) and Fastidious Anaerobe Broth (FAB). This bacterial suspension was used to inoculate three tubes of 2 ml NF broth, with each tube receiving a 20% inoculum volume. Tubes were inoculated at 37 °C for 24 hours under anaerobic conditions, achieved using BD GasPak Anaerobic Sachets (Becton-Dickinson), after which volume of all three tubes were combined. For all bacterial cultures, aliquots of undiluted 24-hour culture were taken for estimation of cell count via optical density reading (OD600). Relationship between optical density and bacterial cell count was determined previously by a constructed standard curve.

### Intravaginal bacterial inoculation

Mice were anesthetized with ketamine (100 mg/kg) and xylazine (10 mg/kg), then atraumatically inoculated intravaginally with 20 ul of either sterile PBS, 10^8^ colony forming units (cfu) *Lactobacillus crispatus* DSM 20584, 10^8^ cfu *Mobiluncus mulieris* DSM 25311, or 10^8^ cfu *Gardnerella vaginalis* ATCC 1418. Mice were left in a supine position for 30 minutes during recovery from anesthesia. All bacterial inoculation studies were done on days 0 and 4 of the experiment. For all bacteria inoculation experiments, two independent investigators were involved as follows: the first investigator prepared all bacteria cultures and was the only person aware of the bacteria culture identities. A second investigator performed intravaginal challenges and downstream analyses blinded.

### Tissue immunohistochemistry

Mouse FGT was harvested and placed in 4% paraformaldehyde (Cat #3190-32 Thermo Fisher Scientific) for 24 hours. FGT tissue was then placed in increasing sucrose solutions, washed in PBS, embedded in OCT compound (Thermo Fisher Scientific), and stored at −80°C for future analysis. Frozen tissues were sectioned using a cryostat at a thickness of 10um and mounted onto glass slides. Slides were washed with PBS/Triton X-100 buffer (Cat #T8787, Sigma-Aldrich) twice for 5 minutes. Tissue sections were pretreated with 50 ul Fc Receptor Blocked (Cat #NB309-5S, Innovex Biosciences Inc) for 30 minutes at RT and subsequently blocked with 2% (w/v) bovine serum, mouse serum (SP-002-VX5, ImmunoReagents Inc), and donkey serum (SP-072-VX10, ImmunoReagents Inc) for 1 hour at RT. Primary antibodies were diluted in blocking buffer and tissues were stained with 1:100 anti-Ly6G (Cat #ab25377, Abcam) and 10 ug/ml anti-Human/Mouse E-cadherin (Cat#AF748, R&D Systems) overnight at 4 °C. Tissues were washed three times with PBS/Triton X-100 solution and incubated with secondary antibodies 1:100 Alexa Fluor 488-conjugated mouse anti-rat immunoglobulin (Ig)G2b (Cat# 172334 Abcam) and 1:200 Alexa Fluor 568-conjugated donkey anti-goat IgG H&L (Cat#Q22079, Thermo Fisher 1:200) for 1 hour at RT. Nuclear staining was performed using Hoechst 33342 (Cat#H1399, Thermo Fisher Scientific) and mounting was done with ProLong Gold antifade mounting media (Cat#P36934, Thermo Fisher Scientific). Vaginal tissue sections were imaged using Zeiss Observer Spinning Disk Confocal microscope and analyzed using the ZEN2 Imaging Software (Oberkochen, Germany).

### FGT permeability assay

Naïve female BALB/c (6–8 weeks old) were anesthetized with ketamine (100 mg/kg) and xylazine (10 mg/kg), then intravaginally inoculated with 50 ug of LY CH, lithium salt (MW = 457.2Da) (Cat# L453, ThermoFisher) in PBS. After 45 minutes, mice were euthanized and the lower FGT excised fixed in 4% (w/v) paraformaldehyde and OCT embedded for analysis. Images were acquired and analyzed blinded to study group using ImageJ software.

### *In vivo* neutrophil depletion

Mice were intraperitoneally (i.p) injected with 500 ug of either isotype IgG2a (clone 2A3, Bio X cell) or anti-Ly6G (clone 1A8, BioXcell) monoclonal antibodies once a week. Neutrophil depletion was confirmed in peripheral blood, spleen, and FGT after 48 hours of antibody injection.

### Sample preparation for mass spectrometry

CVL samples (humans, mice) were processed using protocols described previously^17,92^. Briefly, 25 ug of protein from each sample was digested with trypsin using the filter-aided sample preparation method. For mouse specimens, due to low sample amounts, equal volumes were used at this step. Samples were treated with urea exchange buffer (8M Urea, GE HealthCare; 50 mM HEPES buffer, Sigma) at pH = 8.0. Denatured proteins were transferred to a Nanosep filter cartridge (10 kDA; Pall) and centrifuged at 10,000 g until UEB was removed. Samples were then reduced with 25 mM dithotherol (Sigma) and incubated at RT for 20 minutes, followed by alkylation using 50 mM iodoacetamide (Sigma) for 20 minutes in the dark at RT. Proteins were then digested into peptides using 2 μg of Trypsin enzyme (Promega) at 37°C overnight. Peptides were eluted from the filter cartridge using 50 mM HEPES buffer and were dried using vacuum centrifugation. Peptides were cleaned of salts and detergents using reverse-phase liquid chromatography (high pH RP, Agilent 1200 series micro-flow pump, Water XBridge column) using a step-function gradient prior to analysis by mass spectrometry.

### Mass spectrometry analysis of cervicovaginal fluid

Label-free mass spectrometry analysis of mouse CVL fluid was performed as described previously^17,92^. Briefly, 1μg of peptide from each CVL sample were analyzed using a nano-flow Easy 1000 in-line mass spectrometer with a nano-electrospray ion source at 2.0 kV (Q-Exactive, Thermo Fischer Scientific). Peptides were loaded onto a 100 C_18_-reversed phase Easy-Spray column ES803 (50 cm length, 100 um diameter, 1.8 um particle; Thermo Fisher Scientific). MS1 survey scans were performed using an Orbitrap mass analyzer within the *m/z* range of 300–1700, with a resolution of 70,000 at a *m/z* of 200, and automatic gain control (AGC) target of 3e^6^, with a maximum injection time: 80 ms. The 15 most abundant precursor ions from each survey scan [criteria: isolation width (3 *m/z*), intensity (1e^5^ ions) and charge (+2–5)] were selected for spectra acquisition, and precursor ions were fragmented by high energy collision-induced dissociation (HCD; 28% normalized collision energy). MS2 scans were acquired on the Orbitrap analyzer within a dynamic *m/z* range with a resolution of 17,500 at a *m/z* of 200, and an AGC target of 2e^5^ with a maximum injection time of 100 ms. A reference standard consisting of pooled CVL sample was run every 10 samples to monitor variability in the MS and were used to ensure downstream normalization and data quality. Samples with a median relative protein abundance of 1.5x IQR of the distribution of all samples and did not cluster independently using hierarchical clustering (Euclidean distance metric with complete linkage) were retained for downstream analysis.

Label-free mass spectrometry analysis of human CVL was performed using Orbitrap Eclipse coupled to Easy-nano liquid chromatography (LC) system (Thermo Fisher Scientific), as described previously^17,92^. Peptides (1μg) from each human CVL sample (human) were injected into a nano-flow Easy-nLC 1200 and separated on a C18-reversed phase Easy-Spray column ES903 (50 cm length, 75 um diameter, 2 um particle) (Thermo Fisher Scientific). Peptides were eluted using buffer A (water in 0.1 formic acid) and buffer B (95% acetonitrile in water and 0.1% formic acid) at a constant flow rate of 250 nl/min with a linear gradient from 1% to 35% (buffer B) over 109 min. At 109 minutes, the gradient increased to 90% (buffer B) and was held there for 1 minute and kept at 90% for another 10 minutes. Eluted peptides were electro-sprayed into an Orbitrap Eclipse mass spectrometer with an EASY-Spray ion source (2.0 kV, RF lens: 30%) (Thermo Fisher Scientific). MS1 survey scans were performed using an Orbitrap mass analyzer (m/z range: 375–2000, resolution: 240,000 at a m/z of 200, AGC target: custom at 250% normalized AGC target, maximum injection time: auto mode). Data-dependent acquisitions were acquired using quadrupole-based isolation with top speed mode (cycle time of 1 s), isolation window (0.7 m/z), and charge (+2–7) criteria. Precursor ions were fragmented by high-energy collision-induced dissociation (HCD; 30% normalized collision energy). Fragment ion MS2 scans were acquired on the ion trap analyzer (normal mass range, standard AGC target at 100% normalized AGC target, custom maximum injection time: 30 ms). Reference samples consisting of a pooled CVL sample were run every 10 samples to monitor MS consistency and were used to assess downstream normalization and data quality. Samples with a median protein signal greater than 1.5 times the interquartile range was determined to be outliers and removed from downstream analysis.

### Statistical analysis of human and mouse proteome data

Unpaired two-tailed t tests were performed on log2 normalized data to detect differences between two groups. Significantly different proteins were analyzed through hierarchal clustering in R (NMF package in R, 0.23.0). Biological pathways related to the protein clusters were identified using DAVID gene ontology^93,94^ and Ingenuity Pathway Analysis knowledge bases.

### 16S rRNA sequencing

DNA was extracted from CVL using the DNeasy Blood and Tissue kit (QIAGEN, Inc., Toronto, Canada), with minor modifications to extraction protocol as previously described^95^. The V3–V4 region of the 16S rRNA gene was amplified using primers 341F and 785R^96^. Samples were prepared for and sequenced on MiSeq instrument following manufacturer’s protocol (Illumina Inc., San Diego, USA). The bioinformatics program mothur (v1.39.5)^97^ was used to process sequencing data and assign taxonomy using the naïve Bayesian classifier^98^ and Ribosomal Database Project (RDP) taxonomy database (v16)^99^, as previously describedy^95^.

### Enzyme-linked immunosorbent assay analysis

NE concentration in CVLs were analyzed using a commercially available kit Mouse Neutrophil Elastase/ELA2 (Cat #DY4517-05 R&D). All other cytokines/chemokines were measured by multiplex cytokine arrays by Eve Technologies (Calgary, Canada).

### Image analysis

Time-lapse micrograph images were transformed using Imaris 8.3 (Bitplane) to generate maximum intensity projections and exported as Quicktime movies. Automated 3D tracking of cell centroids was performed for all motility analyses, and subsequent cell track parameters (arrest coefficient, mean displacement) were analyzed using Matlab (Mathworks). Representative images from the lower FGT (vagina and ectocervix) were randomly selected for analysis. Vaginal barrier permeability was quantified by measuring the mean fluorescence intensity of LY dye detected within the region of interest (lamina propria or vaginal epithelial layer) using ImageJ. Neutrophil recruitment into vaginal tissues were also analyzed in a similar manner using ImageJ, by calculating % Ly6G positive signals that cover a normalized tissue area. Vaginal epithelial thickness was determined using E-cadherin staining to create a skeleton of the vaginal epithelium and measuring the distance to its nearest background point along the length of the skeleton using Morpholibj plugin for ImageJ.

### Human study population (THRIVE cohort)

The THRIVE cohort is a prospective study of vaginal mucosal microenvironment of women living in Canada with and without recurrent BV, including those living in Winnipeg and the surrounding area. This study was approved by the Research Ethics Board of the University of Manitoba. This study includes cervicovaginal swab samples from a subset of the THRIVE cohort collected during clinical study visits. For this analysis, we included baseline samples collected from 38 participants, 12 of which were diagnosed with BV at enrollment and 26 without BV by MS/MS. All women were evaluated for STIs at clinical visits and were negative for chlamydia, gonorrhea, and HIV at the time of sampling. A detailed description of participant demographics is provided in the Supplementary Table 1.

### Statistical analysis for mouse studies

Unpaired Student’s t test and Mann-Whitney U-test were used for comparisons of datasets with normal and non-normal distribution, respectively, using Prism 8 (GraphPad). One-way analysis of variance was used to determine statistical differences between the means of three or more independent groups. Median and *p* values from statistical analyses are indicated in each graph. When *p* values were higher than 0.05, differences were considered as not significant.

## Supplementary Material

Supplemental Table1

Proteomics raw data

Proteome pathways

LDvsnLD proteome data

IPA pathways

Supplemental FigureS1toS4

## Data Availability

The data that support the findings of this study are available from the corresponding author upon reasonable request.

## APPENDIX A. SUPPLEMENTARY DATA

Supplementary data to this article can be found online at https://doi.org/10.1016/j.mucimm.2023.04.001.
