# Supplementary material for: Nonoptimal bacteria species induce neutrophil-driven inflammation and barrier disruption in the female genital tract
Source: Mucosal Immunol. Author manuscript; Available in PMC 2026 Jul 27. (PMC13403981; doi:10.1016/j.mucimm.2023.04.001)
Supplement: Supplemental Table1 [file NIHMS2191610-supplement-Supplemental_Table1.pdf]

Supplementary Table 1: THRIVE cohort demographics and clinical data

		Cohort (n=38)		
Variable		*BV negative control group (n=26)*	*BV positive group (n=12)*	p-value*
Age at visit (average, range)		33.1 (20-49)	28.5 (22-48)	0.244097
Ethnicity (n,% within BV group)	White	20 (76.9%)	8 (66.7%)	0.693024
	First Nations	4 (15.4%)	1 (8.3%)	
	Other	2 (7.7%)	2 (16.7%)	
Menstrual phase**	Follicular	5 (19.2%)	2 (16.7%)	0.6352
	Luteal	5 (19.2%)	4 (33.3%)	
	Unknown	16 (61.5%)	6 (50%)	
Microbiome dominance (MS/MS) (n,% within BV group)	*Lactobacillus*	25 (96%)	1 (8.3%)	1.16E-07
	non-*Lactobacillus*	1 (3.8%)	11 (92%)	
Ectropian (n,% within BV group)	Yes	9 (34.6%)	2 (16.7%)	0.44378
	No	17 (65.4)	10 (83.3%)	
Previous STI presence (n,% within BV group)	Yes	12 (46.2%)	6 (50%)	0.872715
	No	13 (50%)	5 (41.7%)	
	Don't know	1 (3.8%)	1 (8.3%)	
Current STI (n,% within BV group)	Yes	0 (0%)	0 (0%)	*NA*
	No	26 (100%)	12 (100%)	
Previously been pregnant (n, % within BV group)	Yes	16 (61.5%)	6 (50%)	0.72486
	No	10 (38.5%)	6 (50%)	
Number of births (average, range)		0.88 (0-3)	0.25 (0-2)	0.074013
Contraceptive use (n, % within BV group)	Copper IUD	3 (11.5)	1 (8.3%)	0.587313
	Hormonal IUD	6 (23.1)	6 (50%)	
	Injection	2 (7.7%)	1 (8.3%)	
	Oral pill	4 (15.4%)	0 (0%)	
	Ring	1 (3.8%)	0 (0%)	
	None	10 (38.5%)	4 (33.3%)	
Taken antibiotics within the past 30 days (n, % within BV group)	Yes	6	4 (33.3%)	0.60849
	No	20	8 (66.7%)	

**p* values calculated using Wilcoxon rank sum test for continuous variables and Fisher’s exact test for categorical variables.

** Menstrual phase determined by hormonal levels and last known menstruation; those participants using hormonal contraceptive are denoted "unknown"
