# Supplementary material for: Nonoptimal bacteria species induce neutrophil-driven inflammation and barrier disruption in the female genital tract
Source: Mucosal Immunol. Author manuscript; Available in PMC 2026 Jul 27. (PMC13403981; doi:10.1016/j.mucimm.2023.04.001)
Supplement: Supplemental FigureS1toS4 [file NIHMS2191610-supplement-Supplemental_FigureS1toS4.pdf]

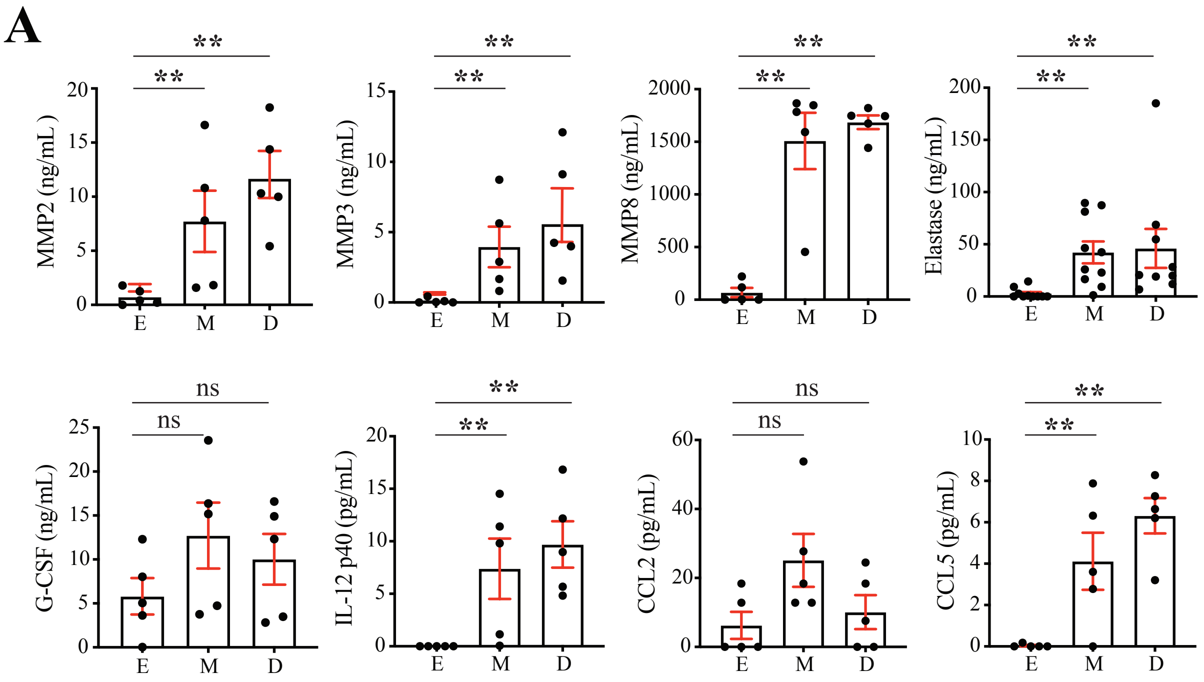

Fig. S1. Cytokine expression in the CVL of Balb/c mice in different estrus phases. (A) Cervicovaginal lavage was collected atraumatically and subjected to multiplex ELISA arrays. The estrus phase was determined by performing vaginal smears. E = estrus, M = metestrus, D = diestrus. Bar graph and red bars indicate Mean +/- SEM values. ** $p<0.01$. ns= not signficiant. Each datapoint represents mean values from triplicate readings, n=5 per group.

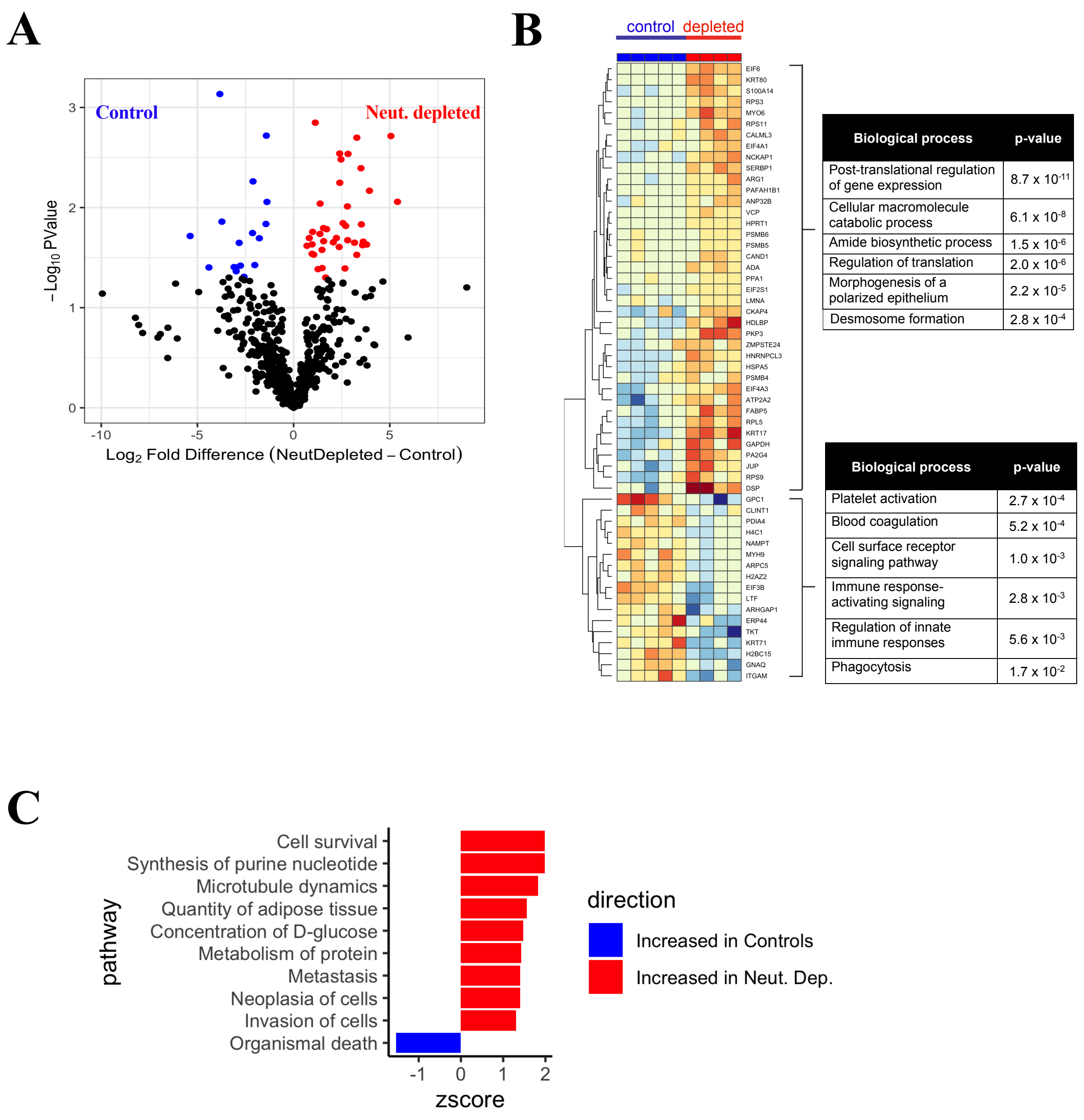

Fig. S2. Proteomic analysis of CVL after neutrophil depletion from FGT. (A) Volcano plot of all proteins identified comparing protein abundance of neutrophil depleted mice (positive values) to those isotypes treated mice (negative values). (B) Hierarchical clustering of differentially abundant proteins identified in CVL of mice injected with either isotype or anti-Ly6G antibody. Proteins that are overabundant are represented in red and those that are underabundant are represented in blue. Samples from isotype treated mice are represented by a blue bar at the top of the heat map and samples from neutrophil depleted mice are represented by a red bar. (C) Canonical pathways significantly enriched in wildtype and neutrophil-depleted mice by IPA. Pathways included have the indicated activation score and p<0.05 right-tailed fisher exact test.

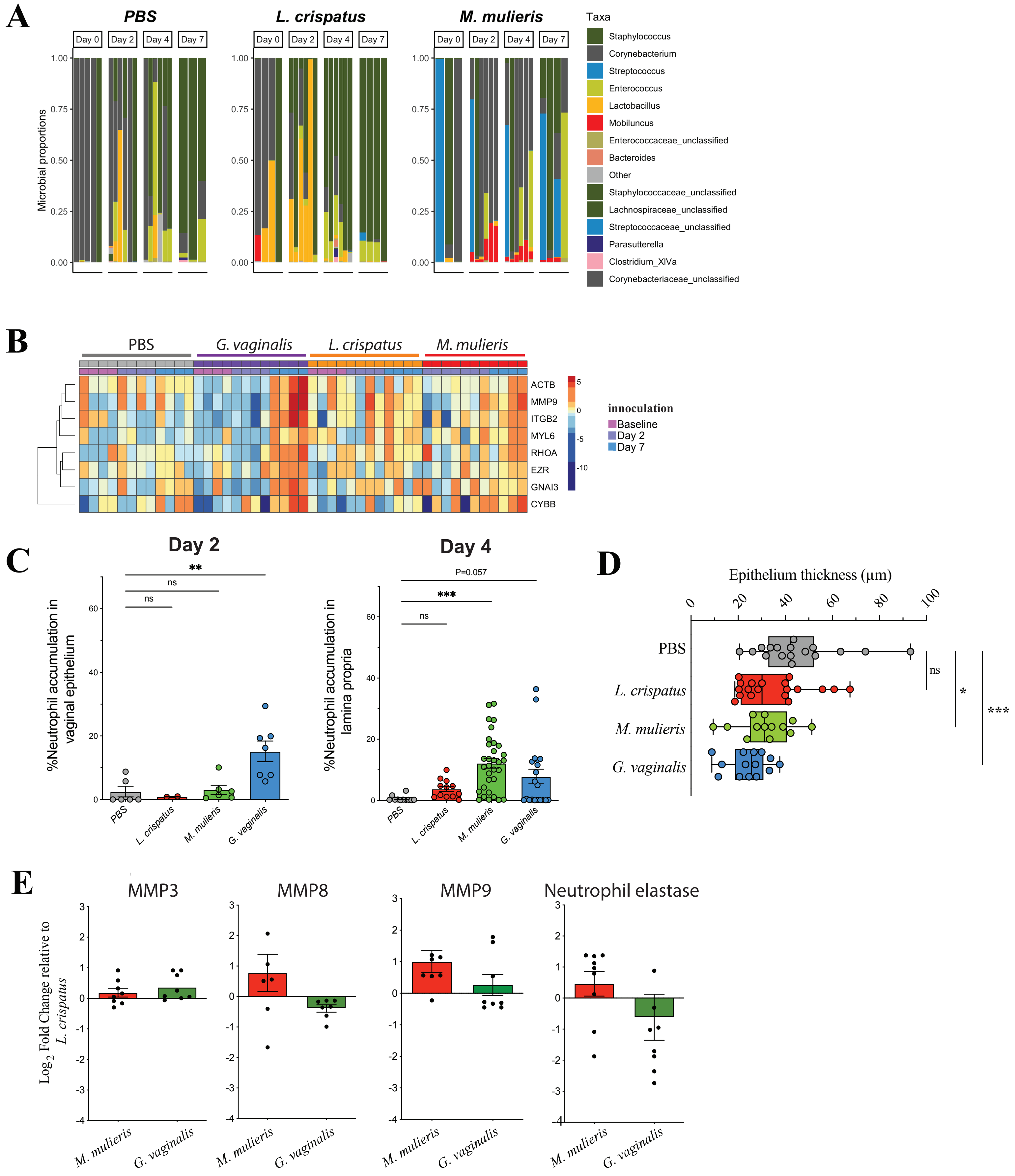

Fig. S3. Neutrophil responses to BV-assocciated bacterial challenge in vivo (A) Time course 16S rRNA analysis of the CVL after intravaginal challenge with the indicated bacterial species. **(B)** Protein expression of neutrophil-related proteins in CVL after bacterial challege. Proteins that are overabundant are represented in red and those that are under-abundant are represented in blue. **(C)** Percentage of vaginal epithelium occupied by Ly6G+ neutrophils at day 2 and 4 post bacterial challenge. **p<0.01. ns=not significant. Mean +/- SEM. **(D)** Vaginal epithelial thickness measurements. *p<0.05; ***p<0.001, ns=not significant, one-way ANOVA. Bar indicates mean values +/- SEM. **(E)** ELISA analysis of proteases in CVL after bacterial challenge. Fold change relative to L. crispatus-challenged mice are shown. Each datapoint indicates mean values from triplicates, pooled data from two independent experiments, n=6-8 mice per group. Mean +/- SEM.

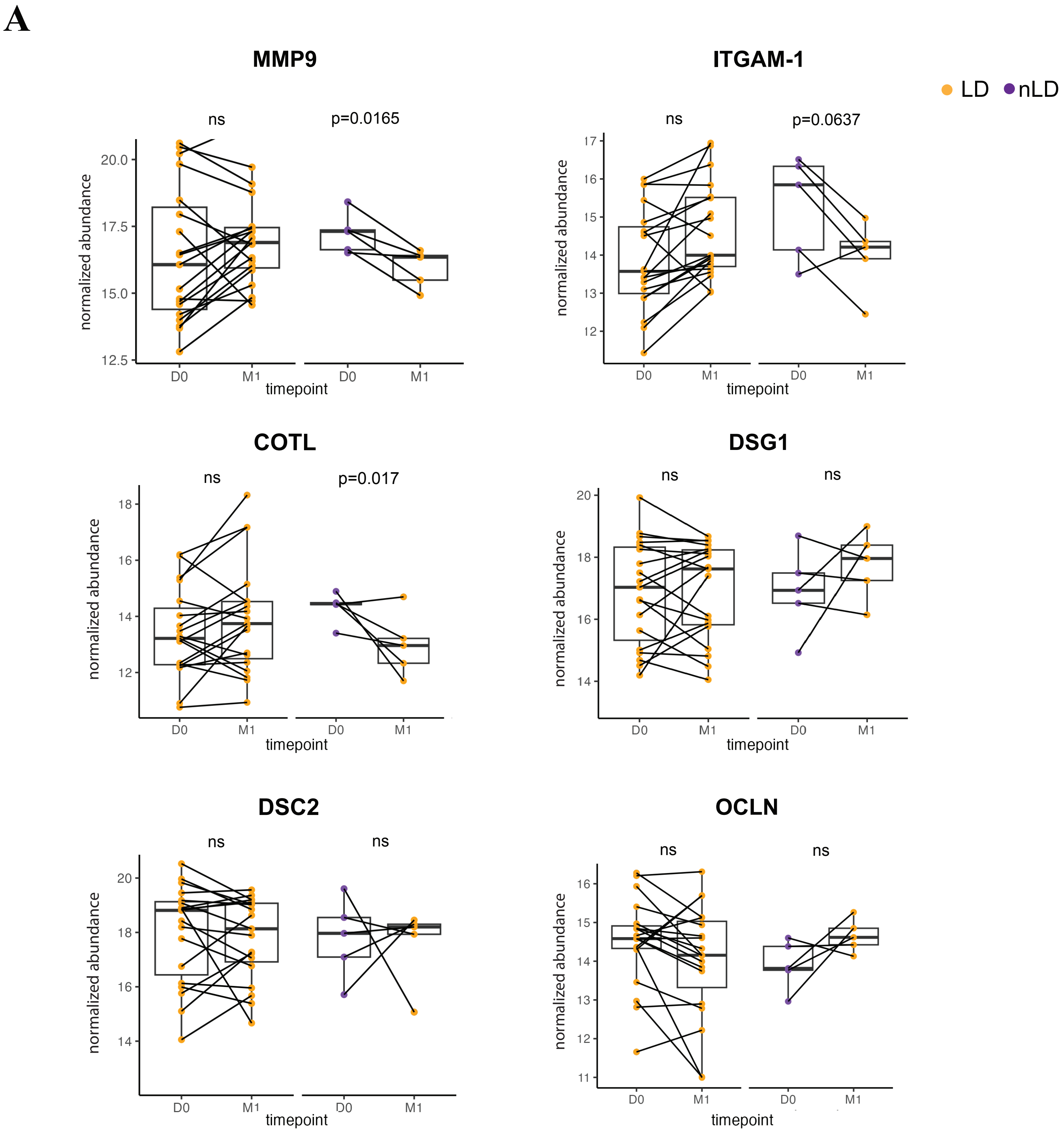

Fig. S4. Neutrophil migration and epithelial barrier disruption protein abundance changes in women who shift from nLD to LD one month after treatment. (A) Protein abundance between LD-to-LD and nLD-to-LD at baseline (D0) and after one month of treatment (M1). Paired two-tailed t-test (Mann-Whitney U-test). ns = not significant.
